# Supplementary material for: HIVprotI: an integrated web based platform for prediction and design of HIV proteins inhibitors
Source: J Cheminform. 2018 Mar 9;10:12. doi: 10.1186/s13321-018-0266-y (PMC5845081; doi:10.1186/s13321-018-0266-y)
Supplement: Supplementary file 2 — Additional file 2. Source code of HIVProtI web server. [file 13321_2018_266_MOESM2_ESM.zip › HIVprotI_Source-code/hivprotI/weka-3-6-6/doc/weka/core/StringLocator.html]

StringLocator


---


|  |  |  |
| --- | --- | --- |
| **Overview** \| **Package** \| **Class** \| **Tree** \| **Deprecated** \| **Index** \| **Help** \| **Weka's home** | |  |
| **PREV CLASS**   **NEXT CLASS** | **FRAMES**    **NO FRAMES**     **All Classes** |  |
| SUMMARY: NESTED \| FIELD \| CONSTR \| METHOD | DETAIL: FIELD \| CONSTR \| METHOD |  |


---


## weka.core Class StringLocator

```
java.lang.Object
  weka.core.AttributeLocator
      weka.core.StringLocator
```

**All Implemented Interfaces:**: java.io.Serializable, java.lang.Comparable<AttributeLocator>, RevisionHandler

---

``` public class StringLocator extends AttributeLocator ```

This class locates and records the indices of String attributes,
recursively in case of Relational attributes. The indices are normally
used for copying the Strings from one Instances object to another.

**Version:**
:   $Revision: 6226 $

**Author:**
:   fracpete (fracpete at waikato dot ac dot nz)

**See Also:**: `Attribute.STRING`, `Attribute.RELATIONAL`, Serialized Form

---

**Constructor Summary**

`StringLocator(Instances data)`
  
          initializes the StringLocator with the given data

`StringLocator(Instances data,
int[] indices)`
  
          Initializes the AttributeLocator with the given data.

`StringLocator(Instances data,
int fromIndex,
int toIndex)`
  
          Initializes the StringLocator with the given data.


| **Method Summary** | |
| --- | --- |
| `static void` | `copyStringValues(Instance instance, boolean instSrcCompat, Instances srcDataset, AttributeLocator srcLoc, Instances destDataset, AttributeLocator destLoc)`             Takes string values referenced by an Instance and copies them from a source dataset to a destination dataset. |
| `static void` | `copyStringValues(Instance inst, Instances destDataset, AttributeLocator strAtts)`             Copies string values contained in the instance copied to a new dataset. |
| `java.lang.String` | `getRevision()`             Returns the revision string. |

**Methods inherited from class weka.core.AttributeLocator**

`compareTo, equals, getActualIndex, getAllowedIndices, getAttributeIndices, getData, getLocator, getLocatorIndices, getType, toString`

**Methods inherited from class java.lang.Object**

`getClass, hashCode, notify, notifyAll, wait, wait, wait`

**Constructor Detail**

### StringLocator

```
public StringLocator(Instances data)
```

:   initializes the StringLocator with the given data

    **Parameters:**: `data` - the data to work on

---


### StringLocator

```
public StringLocator(Instances data,
                     int fromIndex,
                     int toIndex)
```

:   Initializes the StringLocator with the given data.
    Checks only the given range.

    **Parameters:**: `data` - the data to work on: `fromIndex` - the first index to inspect (including): `toIndex` - the last index to check (including)

---


### StringLocator

```
public StringLocator(Instances data,
                     int[] indices)
```

:   Initializes the AttributeLocator with the given data.
    Checks only the specified attribute indices.

    **Parameters:**: `data` - the data to work on: `indices` - the attribute indices to check


**Method Detail**

### copyStringValues

```
public static void copyStringValues(Instance inst,
                                    Instances destDataset,
                                    AttributeLocator strAtts)
```

:   Copies string values contained in the instance copied to a new
    dataset. The Instance must already be assigned to a dataset. This
    dataset and the destination dataset must have the same structure.

    :   **Parameters:**: `inst` - the Instance containing the string values to copy.: `destDataset` - the destination set of Instances: `strAtts` - an AttributeLocator containing the indices of any string attributes in the dataset.

---


### copyStringValues

```
public static void copyStringValues(Instance instance,
                                    boolean instSrcCompat,
                                    Instances srcDataset,
                                    AttributeLocator srcLoc,
                                    Instances destDataset,
                                    AttributeLocator destLoc)
```

:   Takes string values referenced by an Instance and copies them from a
    source dataset to a destination dataset. The instance references are
    updated to be valid for the destination dataset. The instance may have the
    structure (i.e. number and attribute position) of either dataset (this
    affects where references are obtained from). Only works if the number
    of string attributes is the same in both indices (implicitly these string
    attributes should be semantically same but just with shifted positions).

    :   **Parameters:**: `instance` - the instance containing references to strings in the source dataset that will have references updated to be valid for the destination dataset.: `instSrcCompat` - true if the instance structure is the same as the source, or false if it is the same as the destination (i.e. which of the string attribute indices contains the correct locations for this instance).: `srcDataset` - the dataset for which the current instance string references are valid (after any position mapping if needed): `srcLoc` - an AttributeLocator containing the indices of string attributes in the source datset.: `destDataset` - the dataset for which the current instance string references need to be inserted (after any position mapping if needed): `destLoc` - an AttributeLocator containing the indices of string attributes in the destination datset.

---


### getRevision

```
public java.lang.String getRevision()
```

:   Returns the revision string.

    :   **Specified by:**: `getRevision` in interface `RevisionHandler` **Overrides:**: `getRevision` in class `AttributeLocator`
    :   **Returns:**: the revision


---


|  |  |  |
| --- | --- | --- |
| **Overview** \| **Package** \| **Class** \| **Tree** \| **Deprecated** \| **Index** \| **Help** \| **Weka's home** | |  |
| **PREV CLASS**   **NEXT CLASS** | **FRAMES**    **NO FRAMES**     **All Classes** |  |
| SUMMARY: NESTED \| FIELD \| CONSTR \| METHOD | DETAIL: FIELD \| CONSTR \| METHOD |  |


---
